# Nursing Errors in Iranian Nurses: A Mixed Study

**DOI:** 10.1002/hsr2.70904

**Published:** 2025-06-18

**Authors:** Monirsadat Nematollahi, Behnaz Bagherian, Roghayeh Mehdipour‐Rabori

**Affiliations:** ^1^ Department of Pediatric Nursing School of Nursing and Midwifery, Reproductive and Family Health Research Center, Kerman University of Medical Sciences Kerman Iran; ^2^ Department of Medical‐Surgical Nursing Nursing Research Center, Kerman University of Medical Sciences Kerman Iran

**Keywords:** mixed method study, nursing, nursing errors

## Abstract

**Background and Aims:**

Medical errors that result in patient injuries and fatalities are a significant concern in healthcare. Understanding these errors and their contributing factors is essential for enhancing the quality of patient care. This study aimed to investigate nursing errors and the factors that influence them.

**Methods:**

This study employed a mixed‐method approach with a sequential explanatory design and was conducted in Iran from 2022 to 2023. The study consisted of two phases. In the quantitative phase, a cross‐sectional study was conducted in which 200 nurses in Kerman City completed two questionnaires: one on nursing errors and the other on factors influencing nursing errors. In the qualitative phase, the researchers conducted semi‐structured, in‐depth, face‐to‐face individual interviews with 30 nurses.

**Results:**

Two hundred nurses took part in the quantitative phase, and 99% of them reported experiencing at least one nursing error. The highest average score for errors was associated with medication errors (3.40 ± 0.37), followed by care errors (2.85 ± 1.34). The average scores for the most prevalent causes of errors were highest in the organizational dimension (3.8 ± 1.01). A significant positive correlation was found between the average scores of various error dimensions and the average scores of different dimensions related to the causes of errors (*p* < 0.05). Three main categories were identified from the qualitative data: “Nursing errors must be reported,” “Doom of conscience after committing a nursing error,” and “Error in nursing is multifactorial.”

**Conclusion:**

The study revealed a high prevalence of nursing errors, emphasizing the need for proactive measures. Healthcare system managers and decision‐makers must take into account the factors that contribute to nursing errors and respond appropriately to eliminate or reduce them.

## 1 Background

Medical errors have been identified as a significant contributor to patient morbidity and mortality [1]. Studies found that around 400,000 patients who are hospitalized each year suffer from medical errors, and over 200,000 patient deaths are caused by medical errors [2]. Nursing errors are one kind of healthcare errors.

A healthcare error refers to the failure to execute a planned action as intended or the use of an incorrect plan to pursue a desired outcome. These errors are a significant and preventable cause of mortality and mortality among patients, leading to higher healthcare costs and a decrease in the overall quality of the healthcare system [3]. Nursing errors are defined as unintentional mistakes made by nurses that could negatively impact a patient's safety and quality of care [4]. Nurses play a crucial role in ensuring safe patient care as the primary caregivers in hospitals. They spend a significant amount of time with patients, gaining unique insights into their desires, needs, behaviors, and health habits, which makes them important advocates in patient care [5]. However, mistakes can sometimes occur, leading to complications for the patients. Nurses understand that timely reporting can help prevent errors by alerting other nurses [6]. Vaziri et al. [7] demonstrated that nurses in Iran have conducted numerous studies on medical and healthcare errors. Their findings indicated that medication errors were the most prevalent type of error, occurring at a rate of 10%–80%. The majority of these errors were reported in university or teaching hospitals, and the estimated prevalence of medical errors overall was approximately 50% [7].

The causes of nursing errors are largely unknown, but studies have identified several contributing factors, including patient‐related factors, system‐related factors, communication problems, and lack of coordination among healthcare teams [2, 8]. For instance, Zaitoun and colleagues showed that stress, particularly job‐related stress, can impact patient safety [9].

In this study, we employ a mixed methods approach to investigate nursing errors and their contributing factors among Iranian nurses. This design combines both quantitative and qualitative research methods, providing a comprehensive understanding of the issue.

The need for a mixed methods investigation is clear: while quantitative research can reveal the prevalence and characteristics of nursing errors, it often fails to capture the rich contextual factors and underlying reasons. Qualitative methods, particularly in‐depth interviews, allow us to explore the complex experiences and perspectives of nurses, uncovering insights into their thoughts, emotions, and motivations [10]. This dual approach enables us to examine not only the frequency of errors but also the nuances that inform their occurrence, enriching our findings and enhancing the study's validity.

To strengthen our research, we will include detailed information on the recruitment process for both the quantitative surveys and qualitative interviews. This will ensure a representative sample that reflects the diversity of nursing practice in Iran. Additionally, we will specify the methods used to collect quantitative data through surveys and describe the structured approach for conducting qualitative interviews.

By integrating quantitative data with qualitative insights, our mixed methods study aims to provide evidence‐based recommendations that enhance patient safety and improve nursing practice in Iran. We are addressing a critical gap in the literature, ultimately leading to targeted interventions that improve care quality.

## 2 Methods

This study utilized a mixed‐method approach with a sequential explanatory design. The mixed methods approach is a research design that combines both quantitative and qualitative research methods to investigate a research question or phenomenon. It involves collecting and analyzing both numerical data (quantitative) and non‐numerical data (qualitative) within a single study. By integrating these two methods, researchers aim to gain a more comprehensive and nuanced understanding of the research topic [11].

### 2.1 Study Location

The study was conducted in Kerman, Iran. Kerman is the largest province in the southeastern region of the country. Kerman is home to two prominent medical universities, namely Kerman University of Medical Sciences and Azad University. Kerman has four medical educational centers. The research was done in hospitals affiliated with Kerman University of Medical Sciences, located within the province.

### 2.2 Quantitative Phase

In the quantitative phase, a cross‐sectional study was conducted to investigate the types of nursing errors and the factors influencing them among Iranian nurses in Kerman, Iran, from 2022 to 2023. The Strengthening the Reporting of Observational Studies in Epidemiology (STROBE) guidelines were used to ensure that the reporting of observational data was comprehensive and transparent.

The study population consisted of 200 nurses employed in hospitals affiliated with the Kerman University of Medical Sciences, who were selected using a random sampling method. The inclusion criteria stipulated that participants must hold a bachelor's degree or higher in nursing and have a minimum of 6 months of work experience as a nurse in a hospital. The exclusion criteria included incomplete questionnaires and nurses were not directly involved in patient care, such as those with responsibilities other than providing care. Additionally, nurses who assisted with validating the questionnaire were excluded.

Three questionnaires were utilized to gather data on the study objectives: a demographic questionnaire, a nursing error questionnaire, and a questionnaire on factors influencing nursing errors. The researchers distributed these questionnaires to participants while they were at the hospital, asking them to fill them out during their leisure time.

The nursing errors questionnaire was developed because the researchers could not find a suitable existing tool in the literature. It was based on published articles, books, and interviews with expert and practitioner nurses. This questionnaire included 30 questions that assessed different types of errors: care errors (Items 1–11), medication errors (Items 12–18), equipment‐related errors (Items 19–22), and laboratory errors (Items 23–30). Items were rated on a 5‐point Likert‐type scale ranging from *never* (0) to *always* (4), with a total score range of 0–120, where higher scores indicate more errors. Additionally, participants were asked about the number of errors they had made in the last 6 months.

To ensure that the questionnaire captured the distinct factors associated with nursing errors, the researchers conducted an extensive literature review on nursing errors and related factors in English and Persian languages. Existing validated tools that assess medical errors were examined, but none comprehensively covered the specific dimensions related to nursing errors. Consequently, a new questionnaire was tailored specifically to this context, encompassing the relevant dimensions and factors identified in the literature.

The questionnaire was organized into five dimensions: individual factors (12 items), organizational factors (8 items), patient‐related factors (2 items), equipment‐related factors (4 items), and colleague‐related factors (4 items).

To validate the questionnaire, the researchers first conducted a thorough pilot study involving 30 nurses from a similar setting to the main study. This pilot study aimed to evaluate the clarity, relevance, and comprehensiveness of the items, as well as the feasibility of data collection procedures. Following this initial pilot, feedback was used to make necessary revisions, enhancing the clarity and appropriateness of the questionnaire for the target population.

After the revisions were made based on the pilot study, the questionnaire was pre‐tested again with 20 nurses to confirm that the changes improved its effectiveness.

Content validity was assessed using a robust method involving 10 faculty members and 5 clinical nurses from Kerman University of Medical Sciences, yielding a content validity score range of 80%–100%.

Finally, after implementing the revisions and pre‐testing, the researchers calculated a Cronbach's alpha value of 0.718, indicating acceptable reliability for the questionnaire. This value confirms the questionnaire's effectiveness in measuring the intended constructs related to nursing errors.

#### 2.2.1 Data Analysis

Data analysis was conducted using SPSS software version 15 (free version). Descriptive statistics were used to examine the frequency of the data. The Kolmogorov‐Smirnov test was utilized to assess the normal distribution of the variables, and the Pearson correlation coefficient was employed to examine the relationship between types of errors and the factors influencing them. *t*‐Tests and ANOVA were used for data analysis.

### 2.3 Qualitative Phase

The qualitative phase of this study employed the conventional method of qualitative content analysis to analyze the qualitative data obtained from nurses’ experiences with nursing errors and the factors affecting them in hospitals. The conventional method of qualitative content analysis is a widely used approach for analyzing qualitative data in research. It involves systematically and objectively interpreting textual data to identify patterns, themes, and categories. This method follows a series of steps to analyze the data and derive meaningful insights. The conventional qualitative content analysis method allows researchers to systematically examine textual data, identify patterns, and generate insights. It provides a flexible and rigorous approach to analyzing qualitative data, enabling a rich understanding of participants’ experiences, perspectives, and meanings [12, 13, 14].

The researchers adhered to the Consolidated Criteria for Reporting Qualitative Research (COREQ) guidelines to ensure methodological rigor and transparency in the reporting process. The COREQ framework was utilized to guide the development of the study's design, data collection, and analysis processes.

#### 2.3.1 Qualitative Data Collection and Analysis

In the qualitative phase, the researchers conducted semi‐structured, in‐depth, face‐to‐face individual interviews with 30 nurses and head nurses. The interviews were guided by 10 core questions designed to explore participants’ experiences with nursing errors, as well as the factors contributing to these errors. In addition, exploratory probing questions, such as “Could you elaborate further?” were used to elicit more detailed responses.

These participants were selected using a purposive sampling method. A few of the participants were also involved in the quantitative phase, but most were not. The inclusion criteria remained consistent with those used in the quantitative phase. The researcher selected nurses of various genders, ages, and from different units to ensure maximum variation. Interviews lasted an average of 90 min. The sampling took place from March 2022 to April 2023.

The researcher and participants mutually agreed upon the interview locations, with most conducted in a hospital setting. The interviews began with questions focused on participants’ experiences of errors, such as “Have you ever had any errors?” and “What were the factors contributing to these errors?” To facilitate open discussion, additional exploratory and probing questions were incorporated where necessary.

Before conducting the interviews, the researchers established trust with the participants by informing them of the study's objectives. An interviewer not affiliated with the university and unfamiliar with the participants was selected to conduct the interviews. Participants were not required to provide their names and were assured that sharing their experiences would not lead to negative repercussions. As a token of appreciation, participants received a book at the end of the interview.

#### 2.3.2 Data Analysis

In the qualitative phase, the researchers performed conventional content analysis following Graneheim and Landman's approach [15]. Initially, the recorded interviews were listened to four to five times. Subsequently, the complete interviews were transcribed verbatim into a Microsoft Word document. Each transcribed interview served as a unit of analysis, and the same interviewee reviewed and corrected the text. A member of the research team read each finalized text four times, extracting meaningful units to enhance comprehension. These derived meaning units were then condensed and assigned codes. Based on similarities and differences in meaning, the codes were categorized and further classified into subcategories according to their degree of relatedness. Interrelations among subcategories were examined, and the main category was extracted. For example, the phrase “Prevention of errors is necessary” was one of the initial codes that, along with others, contributed to the subcategory “Preventing of errors.” This subcategory, along with two others—“The importance of reporting errors” and “Learning from errors”—collectively formed the main category “errors must be reported.” Throughout each step, the research group members discussed the processes employed and the findings obtained. The final findings were shared with the participants during a meeting, and their final remarks were collected. To facilitate the data analysis process, we utilized MAXQDA software version 10.

#### 2.3.3 The Rigor of the Study

To assess the reliability and validity of the findings, encompassing credibility, confirmability, dependability, and transferability, we employed the criteria proposed by Guba and Lincoln [15, 16]. To ensure data reliability, the researchers established a close and positive rapport with the participants, fostering extensive collaboration. Additionally, we sought input from colleagues and experts and engaged in constant comparisons. To enhance the dependability of the findings, we conducted regular revisions involving experts, participants, and external observers. Personal judgment and experiences were diligently avoided to maintain data confirmability. The researchers made significant efforts to provide thorough explanations of the data to maximize the transferability of the results.

### 2.4 Mixed Methods Integration

The quantitative and qualitative phases were conducted independently of each other. After conducting a statistical analysis of the numerical data and a qualitative analysis of the textual data, the key findings were integrated by merging them at the interpretation and reporting level [10]. The research team reached similar conclusions in two phases. When similar conclusions are derived from combining numerical and textual data, confirming the findings enhances the credibility of the results. Quantitative and qualitative data were presented side by side in a joint tabular display. Inferences and interpretations were made based on the combined findings.

### 2.5 Ethics Approval and Consent to Participate

The Ethics Committee of the Kerman University of Medical Sciences accepted this study with the code IR.KMU.REC.1400.232. All the participants were informed about the study's objectives by the researchers. The participants were assured that their information would remain confidential. Written informed consent was obtained from the participants.

## 3 Results

### 3.1 Socio‐Demographic Data

In the quantitative phase, 200 nurses participated. Among them, 97% (194) held a bachelor's degree, while 3% (6) had a master's degree in nursing. The mean age of participants was 38.4 years (± 8.4), with ages ranging from 23 to 57 years. The majority of the participants were women, comprising 85% (170) of the sample. Additionally, the mean work experience of the participants was 9.2 years (± 6.5). Most participants worked rotational shifts across various wards, including medical, surgical, pediatric, emergency, and intensive care units.

In the qualitative phase, 30 nurses participated; the mean age of the participants was 35.23 ± 4.11. Most of them (22) were women. The mean work experience of the participants was 9.2 years (± 6.5). Most participants worked rotational shifts across various wards, including medical, surgical, pediatric, emergency, and intensive care units.

### 3.2 Quantitative Results

The result showed that 99% (198) of the participants had at least one nursing error in their care. The relationship between the mean total of the medical errors with the demographic characteristics is shown in Table 1. Table 1 shows there was a statistically significant difference between the average score of the nursing errors with a bachelor's degree and postgraduate degree (*p* < 0.02), work experience (*p* < 0.01), workplace (*p* < 0.001), type of shifts work (*p* < 0.01), and employment status (*p* < 0.001).

**Table 1:** The average score of nursing errors in terms of demographic characteristics.

Demographic characteristics	Number	Average score nursing errors (mean ± SD)	*p* value
**Gender**			
Male	194	62.40 ± 6.65	0.18^a^
Female	6	59.13 ± 6.04	
**Marital status**			
Married	160	62.50 ± 5.91	0.24
Single	40	58.98 ± 6.23	
**Education level**			
Bachelor	194	63.10 ± 5.82	0.02^a^
Postgraduate	6	58.28 ± 6.54	
**Work experience**			
< 2 years	35	65.52 ± 5.97	0.01^b^
2–5 years	30	59.34 ± 6.61	
5–10 years	110	56.21 ± 5.13	
> 10 years	25	62.22 ± 6.52	
**Workplace**			
Intensive care unit	63	60.37 ± 6.86	0.001^b^
Department of pediatrics	50	58.87 ± 5.69	
Medical & surgical	87	63.60 ± 4.15	
**Type of shifts work**			
Fixed	17	58.14 ± 7.47	0.01^a^
In circulation	183	63.38 ± 5.77	
**Employment status**			
Temporary employed nurses	37	64.58 ± 4.83	0.01^a^
Registered nurse	163	56.84 ± 4.58	

a *t*‐test.

b ANOVA.

The average score of the nursing errors was 60.74 ± 12.69. The highest errors were related to medication errors 3.40 ± 0.37 and then the care errors 2.85 ± 1.34. Mean laboratory errors were 2.25 ± 0.14, and the equipment errors were 1.15 ± 0.16.

The average score of the factors affecting nursing errors was 89.26 ± 20.40. The highest factors were related to the organizational factors 3.18 ± 0.91 and then the individual factors 2.18 ± 1.02. The average score of the equipment factors and Colleagues factors were 2.02 ± 0.21, 0.47.18 ± 0.08, respectively, and the patient factors were 0.11 ± 0.12.

Table 2 shows the correlation between different dimensions of the errors with different dimensions of the causes of errors using Pearson's correlation coefficient. This table shows that there was a significant positive correlation between the different dimensions of the errors with different dimensions of the causes of errors.

**Table 2:** Correlation between different dimensions of nursing errors with different reasons for nursing errors.

Dimensions of errors	Individual factors	Organizational factors	Factors related to the equipment	Factors related to the patient	Factors related colleagues
Medication errors	0.832*	0.673*	0.154	0.431	0.289
Equipment errors	0.364	0.947*	0.751*	0.142	0.492
Care errors	0.741*	0.364	0.486	−0.268	0.274
Laboratory errors	0.612*	0.541*	0.639*	0.012	0.525

* *p* < 0.05.

The multivariate analysis showed that Organizational and Individual factors were significant determinants of medical errors (Table 3). In the factors affecting nursing errors, results showed that Organizational and individual factors have higher medical errors than other factors.

**Table 3:** Multiple linear analysis on medical errors in association with the factors affecting nursing errors.

Variable	Beta	*T*	*p*
Individual factors	0.697	3.659	0.03
Organizational factors	0.607	2.271	0.003
Factors related to the equipment	0.376	0.224	0.8
Factors related to the patient	0.120	0.083	0.9
factors related colleagues	0.115	0.395	0.7

### 3.3 Qualitative Results

According to data analysis, three main categories were extracted. These categories were “Nursing errors must be reported,” “Doom of conscience after committing a nursing error,” and “error in nursing is multifactorial.”

#### 3.3.1 Nursing Errors Must Be Reported

**Diagram 1:**
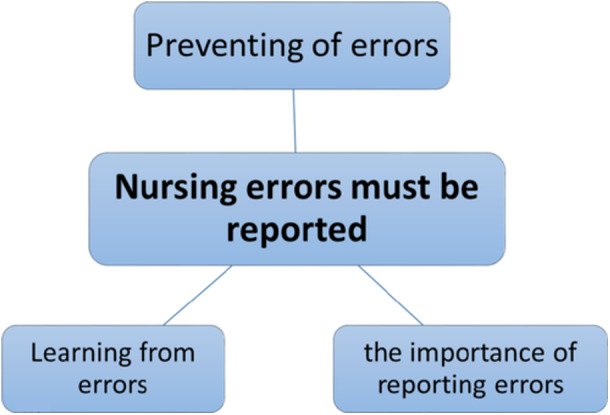
The subcategories for “Errors must be reported.”

The category of reporting nursing errors emerged from all interviews. This category was formed by combining three subcategories “Preventing of errors,” “the importance of reporting errors,” and “Learning from errors” (Diagram 1).

The nurses explained that making errors is inevitable for humans, but nurses must report them because doing so helps other nurses learn from them. Understanding the errors helps nurses make fewer mistakes. Participant number 2 reported, “Nurses should be encouraged to report errors because it will prevent other nurses from making similar errors, and hospital managers will recognize errors and plan to prevent them.” Hospital managers should create a blame‐free reporting system. For example, a participant (N.19) said” Nurses should be able to report incidents without fear of blame or reprisal. She said, “The reporting system should be encouraging, not punitive.” “If it is a punishment, no one will report the error.”

#### 3.3.2 Doom of Conscience After Committing a Nursing Error

The majority of the participants stated that when they noticed they had made a mistake, they were tormented by their conscience. Some of them admitted to crying over their mistakes. A participant said, “When I was a nursing student, I worked in a pediatric unit.” One day, I made a mistake with an injection. After the injection was completed, I realized that it was intended for another patient. I became very upset and cried for an hour. Fortunately, the child was not harmed, but I was tormented by guilt, and I did not return to work until I graduated” (N.7). Some participants believed that conscience prevents many intentional errors. One of the participants said, “During a night shift, I was asleep when I discovered that a patient's dressing was wet and needed to be replaced.” “I wanted to sleep, but my conscience prevented me from sleeping. Finally, I changed the dressing.” (N.27)

#### 3.3.3 Errors in Nursing Are Multifactorial

The main category “Errors in nursing are multifactorial” is obtained from three subcategories “Personal factors” and “Organizational factors.”

The nurses stated that nursing errors have multiple contributing factors. For example, some of the participants explained that individual factors are important in nursing errors. A participant said, “When I was taking care of a patient, my cellphone rang. I have a 3‐year‐old child. The babysitter called from his kindergarten. She told me that my son had stomach pain. I was worried about my son and forgot to give medicine to my patient.” (N.5).

Work experience has also been cited as another important individual factor. A head nurse stated, “Nurses with less work experience make more mistakes than those with more experience.” (N. 10).

These factors consist of individual and organizational agents. The participants referred to both psychological and physical factors, and they said, “If we are calm, we will make little errors. The nurses must be in a good psychological situation state.

Another nurse said, “One day, my child had a fever, and I had to go to the hospital.” “Due to limitations and a shortage of nurses, I had to care for the patients despite my child being sick, but I couldn't do it properly.” (N. 14).

Organizational factors were another important factor in the nursing error. Some nurses stated that managers in hospitals do not help to reduce nursing errors. A nurse said, “The number of staff is low in our unit, and we have to do extra work, and these make us tired. When we get tired, we make more mistakes. The managers do not want to increase our numbers.” (N.18). other nurses said, “We did not have enough training to control the error.” (N.12).

## 4 Discussion

This study aimed to identify nursing errors and their contributing factors in Kerman, Iran, from 2022 to 2023. This study demonstrated that nurses commit errors. Borhani emphasized the importance of safety for patients [17, 18] and nurses must try to reduce errors.

In the present study, most of the nurses reported that they had at least one error. Zirpe in 2020 showed that the prevalence of nursing errors varies across different communities [19]. In the present study, most of the nurses reported that they had at least one error. In recent years, clinical governance and hospital accreditation have been implemented in Iranian hospitals. One of its valuable advantages is the improvement of the culture of reporting nursing errors [20].

This study showed that work experience is an important factor in nursing errors. Jachan et al. [21] demonstrated that work experience reduces error frequencies. Perhaps this is the cause of why less‐experienced nurses make more nursing errors.

The present study showed that nursing errors were less common in the pediatric wards and intensive care units compared to the general wards. Nurses working in pediatric wards pay extra attention to avoiding nursing errors because children are more sensitive to such errors. However, some studies have indicated a high rate of medical errors in pediatric wards [22]. In Iran, expert nurses work in the pediatric and the intensive care units, and maybe that is the reason for low occurrence of nursing errors in these units. In Iran, expert nurses work in the pediatric and intensive care units, which may be the reason for the low occurrence of nursing errors in these units.

The most frequently reported nursing errors in this study were medication errors, which included a lack of attention to drug interactions, incorrect infusion rates, and drug administration without a doctor's order. Other studies also acknowledge that medication errors are the most common errors in hospitals [4].

The findings in qualitative section showed that the most influential factors in the occurrence of medical errors were organizational and individual factors. In this regard, Farokhzadian et al. [23] stated that organizational culture is a main factor in the prevention of medical mistakes, but Brady [24] in his research mentioned that one of the most common reasons for errors was poor clinical performance and lack of information. Wang et al. [25] stated that comprehensive interventions, including organizational, technological, and educational measures, as well as process optimization, can reduce medical errors among nurses. Bari et al. [26] noted that fatigue, lack of experience, and inadequate management were common causes of medical errors. The results of this study, along with previous research, demonstrate that errors in nursing can be attributed to a variety of factors. By identifying these factors and implementing strategies to mitigate them, the occurrence of errors among nurses may be reduced.

This study demonstrated the importance of reporting nursing errors. Diric et al. [27] mentioned that nurses can recognize errors but are reluctant to report them due to fear of the consequences. Mansori et al. [28] reported that the key strategy for addressing obstacles that hinder nurses from reporting adverse events is to create an environment where all nurses feel comfortable reporting errors and the underlying causes truthfully and without fear.

The present study also showed that nurses experience a sense of guilt after committing a nursing error. Robertson et al. [29] reported that nurses and physicians suffer after medical errors and that errors hurt nurses’ health. In this regard, systems and organizations should support nurses and physicians. One way to support them is by providing consulting services.

The current study's findings suggest that by addressing factors that contribute to nursing errors, such errors can be reduced. Therefore, the initial step in decreasing nursing errors is to establish a supportive environment and system that enables nurses to make fewer mistakes. Nurses in the study expressed the importance of reporting errors, indicating that fostering an environment where nurses can openly admit mistakes can promote learning. Additionally, minimizing personal issues among nurses can decrease the likelihood of errors. Healthcare system administrators should focus on addressing medical and nursing errors and implementing strategies to mitigate them, aiming to create circumstances that result in fewer errors by nurses.

## 5 Conclusion

The current study provides detailed information on nursing errors and the factors influencing them in Iran. This knowledge can assist health system managers in identifying necessary reforms to prevent errors in the future. The findings underscore the importance of emphasizing error prevention in pre‐graduate nursing training. Educational programs should incorporate topics such as error identification, reporting mechanisms, and strategies to foster a culture of safety. This emphasis will better prepare nursing students to recognize and address potential errors in clinical practice. Organizations should consider establishing dedicated quality improvement offices tasked with identifying, analyzing, and preventing medical errors. Implementing structured prevention programs and regular training sessions can cultivate a culture of safety that encourages nurses to report errors without fear of repercussion. Additionally, this study has the potential to enhance the training of nurses to mitigate the occurrence of errors. The authors recommend further research and intervention programs that focus on preventing medical errors in hospitals.

The study had limitations such as nurses being hesitant to report errors and providing incomplete questionnaire responses. Additionally, there were fewer postgraduate nurses involved compared to undergraduate nurses. To address these challenges, the researchers worked on establishing trust with the nurses and utilized a combination of research methods.

## Author Contributions

R.M.‐R. and M.N. conceptualized and designed the study. M.N. conducted the fieldwork. M.N. and R.M.‐R. analyzed the data and wrote the first draft. All authors critically revised the manuscript for important intellectual content. B.B. provided direction, particularly on the methodological content. All authors approved the final submitted version. All authors have read and approved the final version of the manuscript. R.M.‐R. had full access to all of the data in this study and takes complete responsibility for the integrity of the data and the accuracy of the data analysis.

## Conflicts of Interest

The authors declare no conflicts of interest.

## Transparency Statement

The lead author Roghayeh Mehdipour‐Rabori affirms that this manuscript is an honest, accurate, and transparent account of the study being reported; that no important aspects of the study have been omitted; and that any discrepancies from the study as planned (and, if relevant, registered) have been explained.

## Data Availability

The data that support the findings of this study are available from the corresponding author upon reasonable request.

## References

[1] A. G. Atanasov , A. W. K. Yeung , E. Klager , et al., “First, Do No Harm (Gone Wrong): Total‐Scale Analysis of Medical Errors Scientific Literature,” Frontiers in Public Health 8 (2020): 558913. 33178657 10.3389/fpubh.2020.558913 PMC7596242

[2] T. L. Rodziewicz , B. Houseman , and J. E. Hipskind , “Medical Error Reduction and Prevention,” In StatPearls [Internet] (Treasure Island, 2021). 29763131

[3] E. Ahsani‐Estahbanati , L. Doshmangir , B. Najafi , A. Akbari Sari , and V. Sergeevich Gordeev , “Incidence Rate and Financial Burden of Medical Errors and Policy Interventions to Address Them: A Multi‐Method Study Protocol,” Health Services and Outcomes Research Methodology 22, no. 2 (2022): 244–252.

[4] N. Alrabadi , S. Shawagfeh , R. Haddad , et al., “Medication Errors: A Focus on Nursing Practice,” Journal of Pharmaceutical Health Services Research 12, no. 1 (2021): 78–86.

[5] S. F. Hawkins and J. M. Morse , “Untenable Expectations: Nurses’ Work in the Context of Medication Administration, Error, and the Organization,” Global Qualitative Nursing Research 9 (2022): 23333936221131779. 36387044 10.1177/23333936221131779 PMC9663611

[6] M. Barkhordari‐Sharifabad and N.‐S. Mirjalili , “Ethical Leadership, Nursing Error and Error Reporting From the Nurses’ Perspective,” Nursing Ethics 27, no. 2 (2020): 609–620. 31331231 10.1177/0969733019858706

[7] S. Vaziri , F. Fakouri , M. Mirzaei , M. Afsharian , M. Azizi , and M. Arab‐Zozani , “Prevalence of Medical Errors in Iran: A Systematic Review and Meta‐Analysis,” BMC Health Services Research 19, no. 1 (2019): 622. 31477096 10.1186/s12913-019-4464-8 PMC6720396

[8] T. L. Rodziewicz and J. E. Hipskind , Medical Error Prevention (StatPearls Publishing, 2020). 29763131

[9] R. A. Zaitoun , N. B. Said , and L. de Tantillo , “Clinical Nurse Competence and Its Effect on Patient Safety Culture: A Systematic Review,” BMC Nursing 22, no. 1 (2023): 173. 37208727 10.1186/s12912-023-01305-w PMC10196295

[10] J. M. Morse , Mixed Method Design: Principles and Procedures (Routledge, 2016).

[11] D. Subedi , “Explanatory Sequential Mixed Method Design as the Third Research Community of Knowledge Claim,” American Journal of Educational Research 4, no. 7 (2016): 570–577.

[12] A. F. Selvi , “Qualitative Content Analysis.” The Routledge Handbook of Research Methods in Applied Linguistics (Routledge, 2019), 440–452.

[13] P. A. Adu , Step‐by‐Step Guide to Qualitative Data Coding (Routledge, 2019).

[14] M. J. Belotto , “Data Analysis Methods for Qualitative Research: Managing the Challenges of Coding, Interrater Reliability, and Thematic Analysis,” The Qualitative Report 23, no. 11 (2018): 2622–2633.

[15] A. J. Kleinheksel , N. Rockich‐Winston , H. Tawfik , and T. R. Wyatt , “Demystifying Content Analysis,” American Journal of Pharmaceutical Education 84, no. 1 (2020): 7113. 32292185 10.5688/ajpe7113 PMC7055418

[16] H. Kyngäs , K. Mikkonen , M. Kääriäinen . The Application of Content Analysis in Nursing Science Research (Springer, 2020).

[17] F. Borhani , A. Abbaszadeh , and R. Rabori , “New Vision for the Dignity: Understanding the Meaning of Patient Dignity in Iran,” British Journal of Medicine and Medical Research 9, no. 2 (2015): 1–11.

[18] F. Borhani , A. Abbaszadeh , and R. M. Rabori , “Facilitators and Threats to the Patient Dignity in Hospitalized Patients With Heart Diseases: A Qualitative Study,” International Journal of Community Based Nursing and Midwifery 4, no. 1 (2016): 36–46. 26793729 PMC4709810

[19] B. Seta , S. Gholap , K. Aurangabadi , et al., “Incidence of Medication Error in Critical Care Unit of a Tertiary Care Hospital: Where Do We Stand?,” Indian Journal of Critical Care Medicine 24, no. 9 (2020): 799–803. 33132563 10.5005/jp-journals-10071-23556 PMC7584841

[20] A. Ranaei , H. A. Gorji , A. Aryankhesal , and M. Langarizadeh , “Investigation of Medical Error‐Reporting System and Reporting Status in Iran in 2019,” Journal of Education and Health Promotion 9 (2020): 272. 33282977 10.4103/jehp.jehp_73_20 PMC7709745

[21] D. E. Jachan , U. Müller‐Werdan , and N. A. Lahmann , “Patient Safety. Factors for and Perceived Consequences of Nursing Errors by Nursing Staff in Home Care Services,” Nursing Open 8, no. 2 (2021): 755–765. 33570279 10.1002/nop2.678 PMC7877149

[22] M. S. Demirtaş , “The Relationship Between Medical Errors Which Commonly Seen in Pediatric Wards With the Mood and Job Motivation of Nurses,” Van Sağlık Bilimleri Dergisi 14, no. 1 (2021): 74–85.

[23] J. Farokhzadian , N. Nayeri , F. Borhani , and M. Zare , “Nurse Leaders’ Attitudes, Self‐Efficacy and Training Needs for Implementing Evidence‐Based Practice: Is It Time for a Change Toward Safe Care?,” British Journal of Medicine and Medical Research 7, no. 8 (2015): 662–671. 26877975 10.9734/BJMMR/2015/16487 PMC4751982

[24] A. P. Brady , “Error and Discrepancy in Radiology: Inevitable or Avoidable?,” Insights Into Imaging 8 (2017): 171–182. 27928712 10.1007/s13244-016-0534-1 PMC5265198

[25] Q. Zhou , H. Wang , J. Jin , et al., “Quality Improvements in Decreasing Medication Administration Errors Made by Nursing Staff in an Academic Medical Center Hospital: A Trend Analysis During the Journey to Joint Commission International Accreditation and in the Post‐Accreditation Era,” Therapeutics and Clinical Risk Management 11 (2015): 393–406. 25767393 10.2147/TCRM.S79238 PMC4354453

[26] A. Bari , R. A. Khan , and A. W. Rathore , “Medical Errors; Causes, Consequences, Emotional Response and Resulting Behavioral Change,” Pakistan Journal of Medical Sciences 32, no. 3 (2016): 523–528. 27375682 10.12669/pjms.323.9701 PMC4928391

[27] H. F. Dirik , M. Samur , S. Seren Intepeler , and A. Hewison , “Nurses’ Identification and Reporting of Medication Errors,” Journal of Clinical Nursing 28, no. 5–6 (2019): 931–938. 30428146 10.1111/jocn.14716

[28] S. F. Mansouri , T. K. Mohammadi , M. Adib , E. K. Lili , and M. Soodmand , “Barriers to Nurses Reporting Errors and Adverse Events,” British Journal of Nursing 28, no. 11 (2019): 690–695. 31188653 10.12968/bjon.2019.28.11.690

[29] J. J. Robertson and B. Long , “Suffering in Silence: Medical Error and Its Impact on Health Care Providers,” Journal of Emergency Medicine 54, no. 4 (2018): 402–409, https://www.ncbi.nlm.nih.gov/pubmed/19666199. 29366616 10.1016/j.jemermed.2017.12.001

